# Cumulative trauma exposure comparison between non-refugee immigrants and locals with psychotic disorder

**DOI:** 10.1192/j.eurpsy.2023.220

**Published:** 2023-07-19

**Authors:** A. Trabsa Biskri, A. Mané, L. González, J. M. Ginés, F. Casanovas, A. Moreno, B. Amann, V. Pérez Sola

**Affiliations:** 1 Universitat Autònoma de Barcelona, Psychiatry and legal medicine department; 2 Institut de Neuropsiquiatria i Addiccions (INAD), Parc de Salut Mar; 3 Institut Hospital del Mar d’Investigacions Mèdiques (IMIM), Psychiatry; 4 Institut de Neuropsiquiatria i Addiccions (INAD), Parc Salut Mar, Barcelona, Spain

## Abstract

**Introduction:**

A significant global increase in immigration has been reported due to humanitarian crisis around the world. Trauma exposure related to migration process is usually multiple and maintained in long-term which could provoke a cumulative effect. Moreover, several meta-analysis describe increased risk for psychosis in immigrant population. Despite this increase, there is a lack of research in non-refugee immigrants specially within those with psychotic disorder.

**Objectives:**

The aim of the study is to describe and compare cumulative lifetime trauma between immigrants and locals with psychotic disorder.

**Methods:**

Patients who have presented, according to DSM-V criteria, one or more non-affective psychotic episodes, were recruited in Acute and Chronic inpatients units at Hospital del Mar (Barcelona) from November 2019 to June 2021, leading to a total sample of 199 patients. Demographic characteristics of patients, clinical data and main pharmacological treatment were recorded through a questionnaire. Database information was completed with electronic medical records. Cumulative trauma Scale was used as instrument to assess lifetime trauma exposure frequency and distress. Comparative analysis was performed with IBM SPSS Statistics (Chicago INC) using Chi-Square Test for qualitative variables and t-Student test for continuous variables. Covariate adjustment with demographic and clinical variables was performed by ANOVA test. Study received local ethics committee approval “CEIC” (No. 2019/8398/I).

**Results:**

From a total of 198 patients, 99 (50%) were immigrants and 99 (50%) locals. Immigrants were exposed on average 3 times more to lifetime traumatic events (16.12) when compared to locals (5.39). Likewise, distress intensity caused by trauma exposure had a mean of 97.13 in immigrants compared to 27.24 in locals. Traumatic events more present in immigrants’ group were “uprooting” (82.8%), “physical abuse” (76.8%), racial discrimination (74.7%), threat of death (74.7%) and life-threatening to close friend (72.2%) and in local group was school failure (42.4%), serious disease (38.4%), accidents (36.4%), physical abuse (36.4%) and interpersonal relationship rejection (36.4%).

**Conclusions:**

According to our results there are important differences in cumulative traumatic events between immigrants and locals with psychotic disorder. Immigrants showed three times more lifetime traumatic events than locals. Likewise, immigrants presented significant higher level of distress caused by lifetime trauma and the nature of traumatic events was more severe. These results should be considered in order to offer better assessment and treatment to this population considering this comorbidity.

**Disclosure of Interest:**

None Declared

